# A Multiplex Assay for Detection of Staphylococcal and Streptococcal Exotoxins

**DOI:** 10.1371/journal.pone.0135986

**Published:** 2015-08-25

**Authors:** Preeti Sharma, Ningyan Wang, Adam S. Chervin, Cheryl L. Quinn, Jennifer D. Stone, David M. Kranz

**Affiliations:** 1 Department of Biochemistry, University of Illinois, Urbana, Illinois, United States of America; 2 ImmuVen, Inc., University of Illinois Research Park, Champaign, Illinois, United States of America; University of Houston, UNITED STATES

## Abstract

Staphylococcal and streptococcal exotoxins, also known as superantigens, mediate a range of diseases including toxic shock syndrome, and they exacerbate skin, pulmonary and systemic infections caused by these organisms. When present in food sources they can cause enteric effects commonly known as food poisoning. A rapid, sensitive assay for the toxins would enable testing of clinical samples and improve surveillance of food sources. Here we developed a bead-based, two-color flow cytometry assay using single protein domains of the beta chain of T cell receptors engineered for high-affinity for staphylococcal (SEA, SEB and TSST-1) and streptococcal (SpeA and SpeC) toxins. Site-directed biotinylated forms of these high-affinity agents were used together with commercial, polyclonal, anti-toxin reagents to enable specific and sensitive detection with SD_50_ values of 400 pg/ml (SEA), 3 pg/ml (SEB), 25 pg/ml (TSST-1), 6 ng/ml (SpeA), and 100 pg/ml (SpeC). These sensitivities were in the range of 4- to 80-fold higher than achieved with standard ELISAs using the same reagents. A multiplex format of the assay showed reduced sensitivity due to higher noise associated with the use of multiple polyclonal agents, but the sensitivities were still well within the range necessary for detection in food sources or for rapid detection of toxins in culture supernatants. For example, the assay specifically detected toxins in supernatants derived from cultures of *Staphylococcus aureus*. Thus, these reagents can be used for simultaneous detection of the toxins in food sources or culture supernatants of potential pathogenic strains of *Staphylococcus aureus* and *Streptococcus pyogenes*.

## Introduction

*Staphylococcus aureus* and group A *Streptococcus pyogenes* secrete a family of pyrogenic toxins including staphylococcal enterotoxins A, B, C (SEA, SEB, SEC), toxic shock syndrome toxin-1 (TSST-1) and streptococcal pyrogenic exotoxins (SpeA, SpeC). A common property of these toxins is that they possess superantigen (SAg) activity that results in overstimulation of the immune system. These T-cell mediated reactions are associated with hyperinflammation and in some cases, organ failure or death. Accordingly, these toxins have been implicated in sepsis, toxic shock syndrome (TSS), infective endocarditis, and skin conditions like atopic dermatitis [1–3].

Some of the staphylococcal toxins are also associated with food poisoning. Accordingly, SEA has often been linked to food poisoning outbreaks [4–9], but staphylococcal enterotoxin B, C, D, E and H have also been shown to be present in some cases [7, 10–13]. In the United States, it has been estimated that *Staphylococcus aureus* mediates approximately 240,000 food-borne illnesses per year [14]. Even if the organisms can be destroyed through a sterilization program, staphylococcal enterotoxins are heat stable and potent at consumption levels as low as a few hundred nanograms [5, 15]. As 20 to 30% of humans carry *S*. *aureus* as a commensal in their skin, its dissemination into foods because of improper handling is a risk factor for food-borne illnesses, compounding the issues associated with improper storage that can allow growth of bacteria and subsequent production of enterotoxins [16]. Like *Staphylococcus aureus*, *Streptococcus pyogenes* colonizes humans and also produces superantigenic toxins including SpeA, SpeC, SMEZ, and SSA that have been linked to conditions like sepsis, streptococcal TSS, scarlet fever, and guttate psoriasis [2, 17–19].

Given the involvement of the superantigen toxins secreted by *Staphylococcus aureus* and *Streptococcus pyogenes* in human illnesses, there has been considerable interest in the development of assays to detect either the toxin genes or proteins. An assay that could detect the presence of multiple superantigens in clinical samples or in the food supply would allow for improved diagnosis in patients with clinical signs of disease or for improved safety of food sources. Here we report the development of a method for multiplex detection of three staphylococcal (SEA, SEB, TSST-1) and two streptococcal toxins (SpeA and SpeC), which could be used for detecting toxins in food, in supernatants derived from blood cultures of *Staphylococcus aureus* and/or *Streptococcus pyogenes*, or potentially directly in clinical samples.

Traditionally, PCR [20–22] and ELISAs using monoclonal and/or polyclonal anti-toxin antibodies [23, 24] have been the method of choice for toxin detection and identification. Anti-toxin antibodies were also used for toxin detection by double immunodiffusion, enzyme linked fluorescence assay (ELFA) [25] and flow cytometry [26]. Although PCR serves as a potential diagnostic method with high sensitivity, it does not provide information about the level of the toxins. For example, it is well known that different SAgs can vary in levels by orders of magnitude depending on transcriptional or post-transcriptional control [27]. In this regard, SEB and TSST-1 have been shown to be highly upregulated at the protein level compared to many other SAgs. Hence, methods that detect and quantitate the level of the pathogenic agent (i.e. the protein) provide a direct correlate of disease caused by that agent. In addition, whereas the bacterial organism can be at low levels or even absent (e.g. due to clearance by the immune system), the toxins are extremely stable and can persist in tissues.

Several investigators have demonstrated that ELISAs can detect staphylococcal toxins with good sensitivity [28–30], although concerns have been raised due to non-specific proteins in bacterial culture supernatants such as protein A that binds to IgG or naturally occurring peroxidases in food [25, 28]. Commercial kits based on ELISA and ELFA using anti-enterotoxin antibody or antibody fragments (Fab) are available with reported sensitivities for staphylococcal enterotoxins A-E of 0.25 to 1 ng/ml. To date, such assays are limited to analysis of individual toxins [11, 31].

In the present study, we took advantage of ultra-high-affinity detecting agents that have been individually engineered for high-affinity binding to SEA, SEB, TSST-1, SpeA and SpeC [32–35]. The detecting agents are the extracellular domains (12 kDa) of the normal receptor for the SAgs, consisting of the variable domain of the T-cell receptor beta chain (Vβ). The collection of five Vβ domains had been engineered, using yeast display and directed evolution, to have ~1,000 to 3 million-fold increases in affinity for the cognate toxins [36, 37]. Here, these five high-affinity Vβ proteins (FL, G5–8, D10V, KKR and HGSM, referred to as Vβ-SEA, Vβ-SEB, Vβ-TSST-1, Vβ-SpeA and Vβ-SpeC, hereafter) were further engineered to contain a C-terminal peptide sequence that allowed site-specific biotinylation. The five biotinylated proteins were immobilized on streptavidin coated, fluorescently labeled-beads to enable high-affinity, specific binding to their respective SAgs. Bound SAgs were detected with commercially available polyclonal reagents, followed by fluorescent-labeled detecting agents. Development of individual assays for each toxin yielded sensitivities in the range of 3 to 6,300 picogram/ml, depending on the toxin.

For comparison, we had recently used direct coupling of the Vβ-SEB to detect 125 pg/ml SEB in different food matrices [31]. We hypothesized that site-specific biotinylation of high-affinity Vβ proteins would allow their immobilization on streptavidin pre-coated beads in an optimal orientation and indeed the biotin approach yielded a 40-fold improvement to 3 pg/ml. To assess the use of the multiplexed assay for simultaneous detection of the staphylococcal SAgs SEA, SEB and TSST-1 in culture supernatants, 18 strains of *Staphylococcus aureus* were analyzed and the levels of the expected toxins were determined quantitatively in all cases.

## Materials and Methods

### Cloning, expression, and purification of high-affinity Vβ proteins in *E*. *coli*

The genes encoding high-affinity Vβ proteins (Vβ-SEA, Vβ-SEB, Vβ-TSST-1, Vβ-SpeA and Vβ-SpeC) were cloned with a N-terminal 6X-His tag and a C-terminal Avitag (Avidity, LLC) in pET28a expression vector (Novagen) for protein expression in *E*.*coli* BL21 (DE3) cells as inclusion bodies. The proteins were refolded (by slow dilution) from denatured inclusion bodies, followed by affinity purification with Ni agarose resin (Qiagen) and HPLC (Biocad Sprint) using a Superdex 200 (GE Healthcare) size exclusion column as described previously [33].

### *In vitro* biotinylation of purified Vβ proteins

Monomeric fractions of refolded proteins were biotinylated at the specific lysine residue in the Avitag sequence (GGGLNDIFEAQKIEWHE), using BirA enzyme (Avidity, LLC) overnight at 4°C. Excess biotin was removed and biotinylation buffer was exchanged to 1X PBS, pH = 7.4 using Zeba spin columns (Thermo Fisher Scientific Inc, IL, USA). Biotinylation was assessed by incubating the proteins with streptavidin and analyzing their change in mobility (“gel-shift”) on 4–20% polyacrylamide gels.

### Toxin binding titration with Vβ-immobilized beads in singleplex format

Biotinylated, high-affinity Vβ proteins (Vβ-SEA, Vβ-SEB, Vβ-TSST-1, Vβ-SpeA or Vβ-SpeC) were immobilized on fluorescent polystyrene beads that contained covalent-linked streptavidin (Spherotech, Inc.). In preliminary studies to assess the binding capacity of streptavidin beads, different amounts of biotin-Vβ were immobilized on the beads to determine optimum density of each biotin-Vβ on the bead surface that yielded minimum background and maximum sensitivity. Different dilutions of polyclonal antibodies were also tested to determine optimum concentrations used for detection of the captured toxin. After biotin-Vβ was immobilized on beads, unbound protein was removed by washing and unoccupied biotin binding sites were blocked by incubation with 0.75 mM free biotin. After blocking with ~1000-fold excess biotin compared to the biotin-binding sites available on the beads, Vβ immobilized-beads were washed and incubated with various concentrations of recombinant toxins (SEA, SEB, TSST-1, SpeA or SpeC) (Toxin Technology, Inc.). After washing, beads were incubated with a 1:100 dilution of rabbit anti-toxin polyclonal antibodies (anti-SEA, Sigma-Aldrich; anti-SEB, Pierce; anti-TSST-1, Abcam; anti-SpeC, Abcam). Anti-SEB was found to contain antibodies that cross-reacted with the structurally similar SpeA, hence it was used for SpeA detection. After washing, the biotin-Vβ beads were incubated with 1:200 dilution of goat-anti rabbit IgG(H+L) labelled with Alexa fluor 647 (Life Technologies). After 45 minutes, the beads were washed and bound toxins were detected by flow cytometry as an increase in fluorescence signal in the appropriate fluorescence channel (λ_emission_ of Alexa fluor 647 = 688 nm), compared to fluorescence emitted by the beads to which no toxin was added. Fluorescence emitted by the beads was measured using Accuri C6 flow cytometer (BD Biosciences), at room temperature. 1X PBS + 2% BSA was used for diluting all reagents and washing the beads. All assays with the five different Vβ proteins were quantitatively validated in singleplex format prior to use in multiplex format.

### Toxin detection with mixtures of Vβ immobilized-beads in multiplex format

Biotinylated, high affinity Vβ proteins (Vβ-SEA, Vβ-SEB, Vβ-TSST-1, Vβ-SpeA and Vβ-SpeC) were immobilized on individual fluorescent polystyrene beads (P12, P10, P6, P2 and P8 respectively, each having unique fluorescence in the FL-1 channel (λ_emission_ of beads = 525 nm) that contained covalently linked streptavidin (Spherotech, Inc.). After blocking with excess biotin, the Vβ immobilized-beads were washed, combined and incubated with various recombinant toxins at different concentrations or culture supernatants from strains of *Staphylococcus aureus*. After 60 minutes, beads were washed and incubated with a mixture of rabbit anti-toxin polyclonal antibodies (1:100 dilution), followed by washing and incubation with 1:200 dilution of goat-anti rabbit IgG(H+L) labelled with Alexa fluor 647 (Life Technologies). After 45 minutes, the beads were washed and bound toxins were detected by flow cytometry as an increase in fluorescence signal in the appropriate fluorescence channel (λ_emission_ of Alexa fluor 647 = 688 nm), compared to the fluorescence emitted by the beads to which no toxin was added. The identity of the specific toxin was established by determining the fluorescent bead(s) (in the spectrally distinct, FL-1 channel; λ_emission_ = 525 nm). Fluorescence emitted by the beads was measured using Accuri C6 flow cytometer (BD Biosciences), at room temperature. 1X PBS + 2% BSA was used for diluting all reagents and washing the beads.

### Culture of *S*. *aureus* strains

*S*. *aureus* isolates used in this work are listed in Table 1. Strains were purchased from the Network on Antimicrobial Resistance in *Staphylococcus aureus* (NARSA), now BEI Resources, NIAID, NIH (http://www.beiresources.org/). Information about presence or absence of SAg genes, and/or proteins in these strains was obtained from GenBank/NARSA/other investigators [38, 39], as indicated in Table 1. Strains were grown by Micromyx, LLC (Kalamazoo, MI) in Todd Hewitt Broth (THB) for 16–20 hours with shaking at 200 rpm at 37°C. After incubation, cultures were centrifuged at 5,000 x g for 10 minutes to pellet the cells. The supernatant was passed through a 0.2 μm filter to obtain cell free supernatant. Supernatants were stored at-20°C, prior to using in multiplex assays.

**Table 1:** Summary of sources of *Staphylococcus aureus* strains analyzed. Where available, information about presence or absence of SAg genes, and/or proteins, is shown. These were also compared with the SAg expression profiles of each strain, as determined by multiplex assay in the present work. “+” represents fluorescence signal above the background in multiplex assay (also shown in Fig 6), indicating the presence of toxin indicated.

Strain (NARSA nomenclature/aliases)	Source of isolate^a^	Presence or absence of SEA, SEB, or TSST-1 gene(s) or protein(s), from GenBank^b^/NARSA^c^/other investigators^d^	Presence of SAg detected by Multiplex assay (Fig 6)		
			SEA	SEB	TSST-1
NRS1/ATCC700699/Mu50	Pus and debrided tissue at surgical incision in sternum of 4 month-old infant, Japan	SEA gene^+^ (GenBank); TSST-1 gene^+^ (GenBank)	+		+
NRS2/ATCC700698	Not reported	Not reported	+		+
NRS22/HIP07930/USA600/99758	Bloodstream of an adult female ICU patient, US	SEA gene^-^ (NARSA); SEB gene^-^ (NARSA); TSST-1 gene^-^ (NARSA)			
NRS70/N315/USA100	Pharyngeal smear of a patient, Japan	TSST-1 gene^+^ (GenBank)			+
NRS100/COL	Archaic methicillin resistant *S*.*aureus* (MRSA) strain, UK	SEB gene^+^ (GenBank)		+	
NRS111/TKI913/FRI913	Food associated with a staphylococcal food poisoning outbreak, US	SEA gene^+^ (NARSA); TSST-1 gene^+^ (NARSA); SEA gene^+^ (PCR) [39]; TSST-1 gene^+^ (PCR) [39];	+		+
NRS112/MN8	Patient with menstrual TSS, US	SEA gene^+^ (PCR) [38]; TSST-1^+^ [38];	+		+
NRS113/MNDON	Not reported	Not reported		+	
NRS114/MNHOCH	Not reported	SEB gene^+^ (PCR) [39];		+	
NRS382/USA100	Bloodstream sample, US	Not reported			
NRS384/USA300	Isolated from wound, US	Not reported			
NRS387/USA800	Isolated from wound, US	SEB gene^+^ (NARSA)		+	
NRS651/CA-409	Isolated from peritoneal fluid, US	TSST-1 gene^+^ (NARSA)	+		+
NRS667/CO-71	Not reported	Not reported			
NRS695	Not reported	Not reported	+		
NRS701/MN-082	Not reported	Not reported			+
NRS725/OR-25	Not reported	Not reported			
NRS741/TN90	Not reported	Not reported			

^**a**^Sources of the isolates were described by NARSA or ATCC (American Type Culture Collection).

^**b**^Information about presence or absence of SAg genes was obtained by performing BLAST analysis on genome sequences available in GenBank (NIH genetic sequence database).

^**c**^Information about presence or absence of SAg genes was obtained as annotations from NARSA website.

^**d**^Information about presence or absence of SAg genes was established by other investigators (references indicated).

## Results

### Design of the bead-based two-color flow cytometry assay

**Fig 1:**
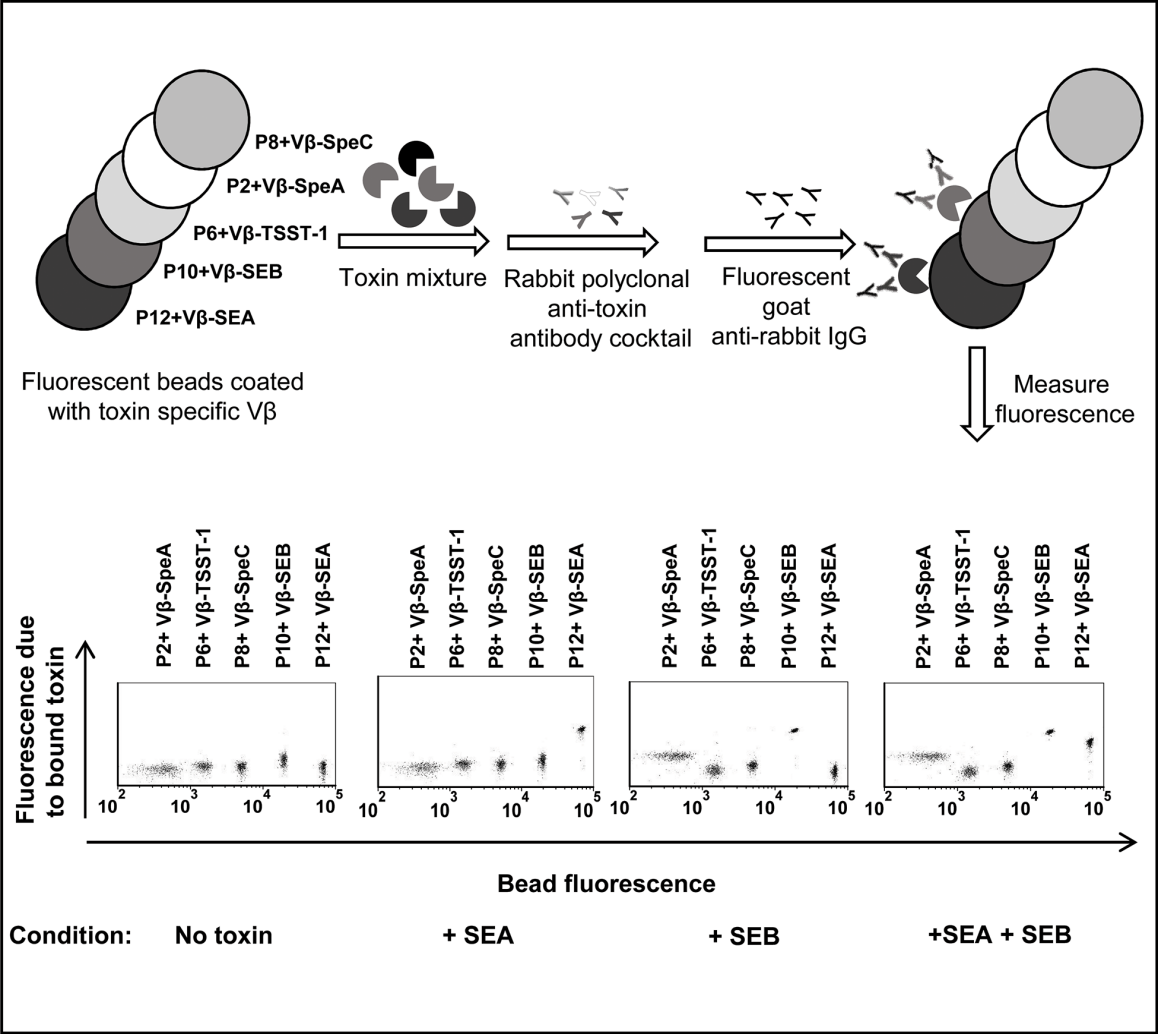
Schematic diagram and representative flow cytometry data of the multiplex assay. Biotinylated, high-affinity Vβ proteins (Vβ-SEA, Vβ-SEB, Vβ-TSST-1, Vβ-SpeA and Vβ-SpeC) were immobilized on streptavidin-coated fluorescent beads (P12, P10, P6, P2 and P8 respectively, each having unique fluorescence in the FL-1 channel, λ_emission_ = 525 nm). For detecting the presence of one or more toxins (SEA and/or SEB in this example), a mixture of Vβ immobilized-beads was added to an unknown sample. Toxins captured by the Vβ-immobilized beads were detected by rabbit polyclonal anti-toxin antibodies followed by goat-anti rabbit IgG labeled with Alexa fluor 647 (λ_emission_ = 688 nm in FL-4 channel). Scatter plots in the absence or presence of toxin(s) are shown in the lower panel. An increase in fluorescence emitted by the beads in the FL-4 channel (Y-axis) indicates the presence of toxin(s) in the unknown sample. Since each class of beads emits distinct fluorescence in FL-1 channel (X-axis), identity of the toxin present is established by identifying the specific, Vβ-immobilized bead(s) undergoing an increase in fluorescence in the FL-4 channel (Y-axis). In the presence of SEA, only the P12 beads (immobilized with Vβ-SEA) exhibited an increase in fluorescence along Y-axis. In the presence of SEB, P10 beads (immobilized with Vβ-SEB) as well as P2 beads (immobilized with Vβ-SpeA) exhibited increases in fluorescence along Y-axis, due to low affinity of Vβ-SpeA toward SEB. Accordingly, in the presence of SEA and SEB, Vβ-immobilized P12, P10 and P2 beads exhibited increases in fluorescence along Y-axis.

The bead-based multiplex assay took advantage of a panel of streptavidin coated polystyrene beads (P2, P6, P8, P10, P12), each with distinct fluorescence intensities (Fig 1). Each type of bead was used to immobilize a single, purified high-affinity Vβ protein with covalently linked biotin. The high-affinity Vβ proteins served to “capture” the specific toxin against which they were engineered. The Vβ-SpeA and Vβ-TSST-1 also have low affinities for the structurally related SAgs SEB and SpeC, respectively [32–35]. The “captured”, bead-bound toxins were detected by use of rabbit polyclonal anti-toxin antibodies followed by a fluorescent-labeled (Alexa fluor 647) goat-anti rabbit IgG.

The use of both the single Vβ agents and the polyclonal reagents each provide an opportunity for specificity in the assays. Two-color flow cytometry allowed the fluorescence emitted by the fluorophore within each class of beads (FL-1 channel) to be distinguished from the fluorescence due to the bound toxin (FL-4 channel). This design allowed identification of the toxin by association with the specific Vβ-immobilized bead, and quantification of the amount of toxin by the level of fluorescence from the Alexa fluor 647 reagent (Fig 1, lower panel).

### Biotinylation of high-affinity Vβ proteins

**Fig 2:**
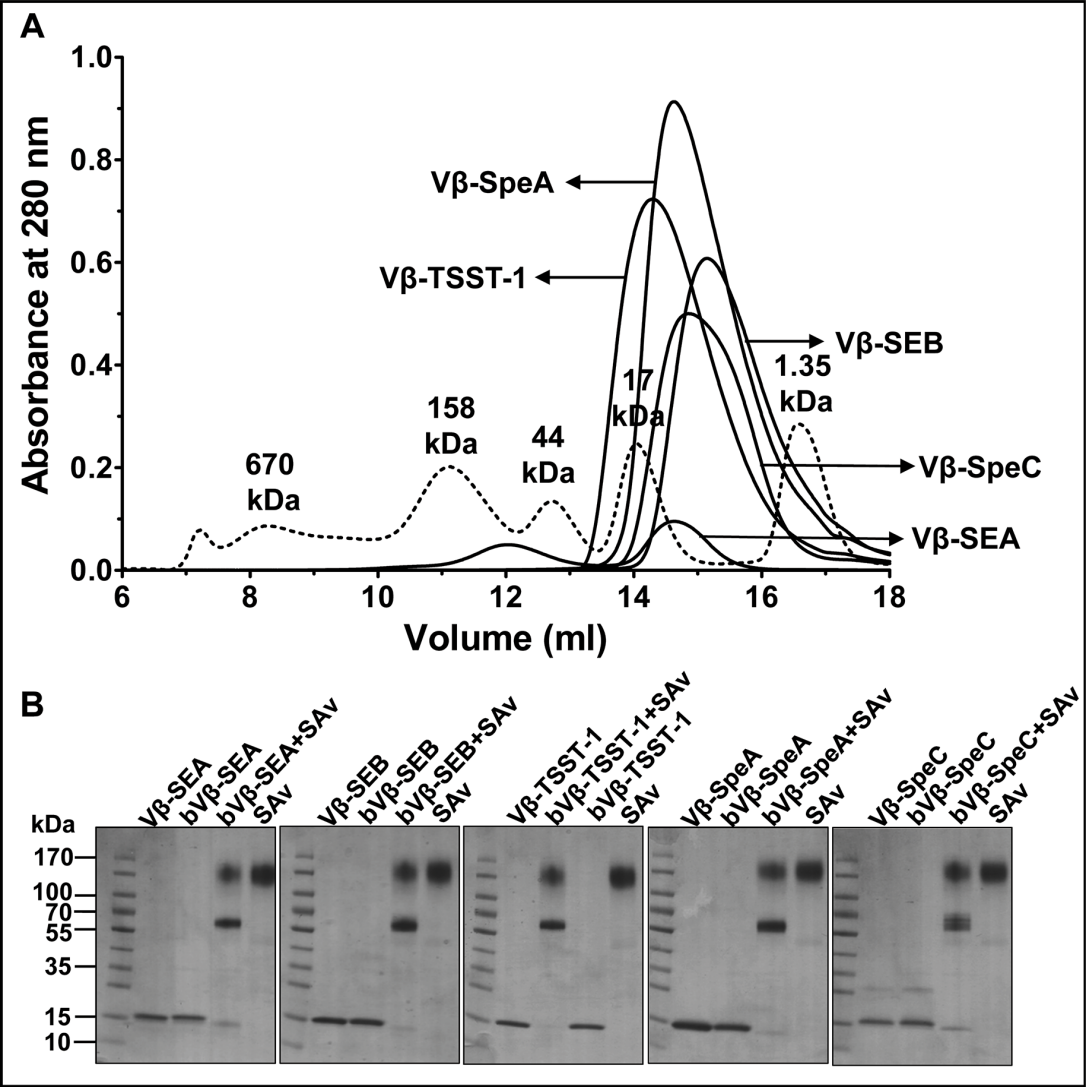
Expression and biotinylation of high-affinity Vβ proteins. (A) Purification of monomeric fractions of refolded, high-affinity Vβ proteins by size-exclusion chromatography. Dashed line indicates the molecular weight standards. (B) Gel-shift assay for monitoring biotinylation of high-affinity Vβ proteins (Vβ). Disappearance of biotinylated-Vβ (bVβ) (~15kDa band) in the presence of streptavidin (SAv) was indicative of biotinylation.

In order to generate bead-based arrays of high-affinity capture agents, the genes encoding five high-affinity Vβ proteins were cloned with an N-terminal 6X-His tag for purification and a C-terminal ‘Avitag’ sequence (GGGLNDIFEAQKIEWHE) for enzymatic linkage of biotin to lysine. The proteins were expressed in *E*. *coli* and purified from inclusion bodies after *in vitro* refolding. Ni-affinity purified proteins were subjected to S200 gel filtration yielding a substantial fraction of monomeric Vβ protein in each case (~15 to 18kDa apparent mobility) (Fig 2A). The monomeric fractions were concentrated and biotinylated *in vitro* by addition of BirA ligase and 100 μM biotin.

To estimate the fraction of Vβ protein that was biotinylated, we used a gel-shift assay that takes advantage of the observation that biotin remains bound to streptavidin (SAv) even under SDS-PAGE conditions. Over 80% biotinylation of each Vβ protein was observed, as evidenced by the disappearance of the majority of the Vβ-biotin species (~15 kDa band). The emergence of a molecular weight species of approximately 70 kDa was presumably due to the migration of the Vβ-biotin (~15 kDa) complexed with tetrameric streptavidin (~55 kDa), which is structurally more compact (and hence migrates faster on SDS-PAGE) than the higher molecular weight bands (100–130 kDa) which likely correspond to unbound streptavidin tetramers, or aggregates of streptavidin tetramers [40, 41].

To further verify that the biotinylated Vβ proteins retained the ability to bind to their respective toxins, the proteins were incubated with streptavidin-coated beads, and the washed Vβ immobilized beads (Vβ-SEA, Vβ-SEB, Vβ-TSST-1, Vβ-SpeA and Vβ-SpeC) were incubated with 50 nM toxin (SEA, SEB, TSST-1, SpeA and SpeC respectively), followed by washing and incubation with 1:100 or 1:1000 dilutions of anti-toxin antibody (anti-SEA, anti-SEB, anti-TSST-1, anti-SpeA and anti-SpeC respectively). After washing, the beads were incubated with 1:100 dilution of goat-anti rabbit IgG(H+L) labelled with Alexa fluor 647. In each case, binding to toxin was detected as an increase in fluorescence of the washed beads in the appropriate fluorescence channel (S1 Fig).

### Sensitivity of toxin detection in singleplex assays

**Fig 3:**
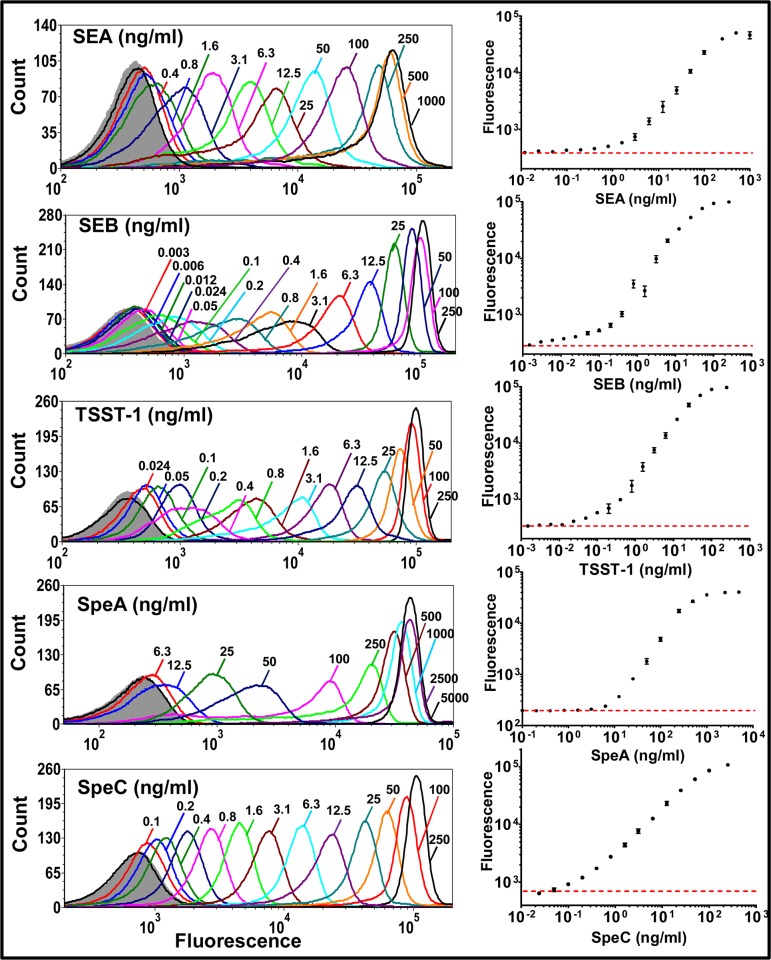
Sensitivity of toxin detection by Vβ-immobilized beads in singleplex assays. Each biotinylated, high-affinity Vβ protein (Vβ-SEA, Vβ-SEB, Vβ-TSST-1, Vβ-SpeA and Vβ-SpeC) immobilized on individual streptavidin-coated fluorescent beads was subjected to cognate, toxin binding titration (SEA, SEB, TSST-1, SpeA and SpeC respectively). Toxins captured by the Vβ-immobilized beads were detected with rabbit polyclonal anti-toxin antibodies (anti-SEA, anti-SEB, anti-TSST-1, anti-SEB and anti-SpeC respectively) followed by goat-anti rabbit IgG labeled with Alexa fluor 647. Flow cytometry histograms for each binding titration are shown, with fluorescence arising due to toxin binding on X-axis. Fluorescence in the absence of toxin is represented by gray (filled) trace on each histogram. Median fluorescence units from the histograms were used to generate binding curves, shown on the right. Red, dashed line indicates fluorescence in the absence of toxin. Data shown are representative of experiments performed in triplicate.

In order to determine the specificity and sensitivity of the flow cytometry-based assays using the biotinylated high-affinity Vβ proteins (Vβ-SEA, Vβ-SEB, Vβ-TSST-1, Vβ-SpeA and Vβ-SpeC), each Vβ protein was tested in a singleplex assay (Fig 3). Each assay showed a dynamic range of over three orders of magnitude for determining concentrations of the specific toxin. The range spanned the picogram per ml level to hundreds of nanograms/ml level. As determined from the low end of the flow cytometry histograms, the assay detection limits were approximately 400 pg/ml SEA, 3 pg/ml SEB, 25 pg/ml TSST-1, 6000 pg/ml SpeA and 100 pg/ml SpeC (Fig 3).

We compared the sensitivities of toxin detection in the flow-cytometry singleplex assays with a standard capture ELISA (where the Vβ proteins were adsorbed to 96-well plates, and detection was with the same polyclonal reagents as in the flow cytometry assay). Sensitivities were calculated as the lowest toxin concentration at which the median fluorescence intensity or absorbance (measured at 450 nm) was at least 3 standard deviations above the negative control. The detection limits in the capture ELISAs were 4 to 80 fold higher than in the flow cytometry assay (S2 Fig; Table 2). We believe that the enhanced sensitivity of the flow-based assay is in part due to the greater surface density of properly arrayed Vβ domains on the beads compared to the adsorption of Vβ proteins, some in a non-binding configuration, to the wells of a 96-well microtitre plate. It is worth noting the lower sensitivity of SpeA detection in both the bead-based approach and in ELISAs. We believe that this could have to do with the fact that we were unable to identify a suitable anti-SpeA polyclonal preparation and thus used a cross-reacting anti-SEB antibody preparation.

**Table 2:** Comparison of sensitivities of toxin detection in capture ELISA versus a bead-based, singleplex, flow cytometry assay.

Toxin	Sensitivity of detection by capture ELISA (ng/ml)	Sensitivity of detection by singleplex, flow cytometry assay (ng/ml)	Fold improvement in sensitivity in bead-based, singleplex, flow cytometry assay
SEA	2.5–10	0.4	6–25
SEB	0.25	0.003	83
TSST-1	0.1–0.25	0.025	4–10
SpeA	25–100	6.3	4–16
SpeC	1–2.5	0.1	10–25

### Detection and quantitation of multiple toxins in a multiplex assay

**Fig 4:**
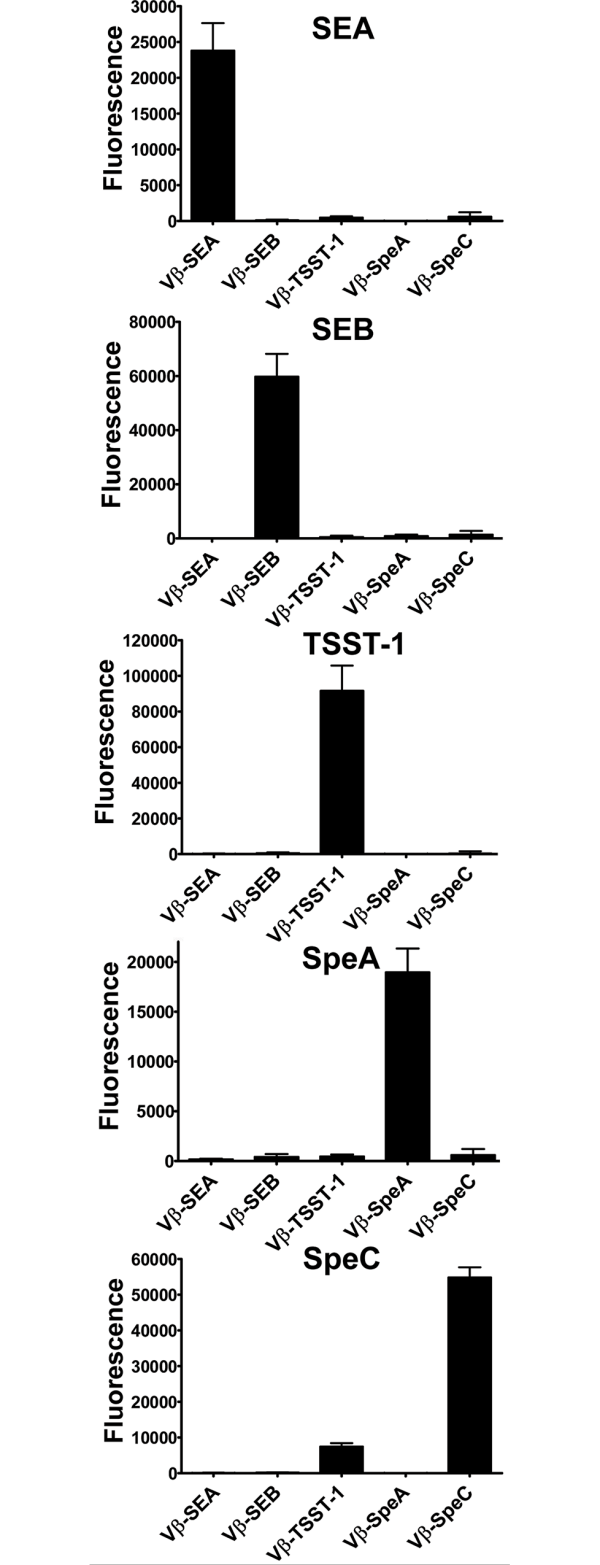
Multiplex assay in the presence of one toxin. Solutions containing 50 ng/ml SEA, 10 ng/ml SEB, 10 ng/ml TSST-1, 50 ng/ml SpeA or 10 ng/ml SpeC were tested in multiplex assays. Fluorescence emitted by each Vβ-immobilized bead due to toxin binding, was plotted on bar graphs shown. In all cases, the toxins were detected by the beads immobilized with the high-affinity Vβ engineered for that toxin. The error-bars represent standard deviations from two independent experiments. Similar data (but with higher background) were obtained in other experiments where unoccupied sites on biotin-Vβ immobilized-beads were not blocked with excess biotin (S4 Fig).

In order to detect and quantitate multiple toxins in the same sample, a mixture of Vβ-immobilized beads were incubated with specific toxins and bound toxins were detected with a mixture of the polyclonal anti-toxin antibodies (Fig 4). In this assay format, SEA, SEB, TSST-1, SpeA and SpeC were detected by their Vβ-SEA, Vβ-SEB, Vβ-TSST-1, Vβ-SpeA and Vβ-SpeC probes, respectively (Fig 4). As expected, the Vβ-SpeA exhibited some cross-reactivity with SEB at concentrations higher than 10 ng/ml (S3 Fig); [34]. The Vβ-TSST-1 also cross-reacted with SpeC at concentrations higher than 1 ng/ml (S3 Fig), probably because TSST-1 and SpeC both bind to the same human Vβ region, Vβ2.1, and in fact the Vβ-TSST-1 and Vβ-SpeC high-affinity mutants were both engineered from the human Vβ2.1 mutant EP-8 that binds to both SAgs [32]. Similarly, we also observed that Vβ-SpeC cross-reacted with TSST-1 at concentrations higher than 10 ng/ml.

**Fig 5:**
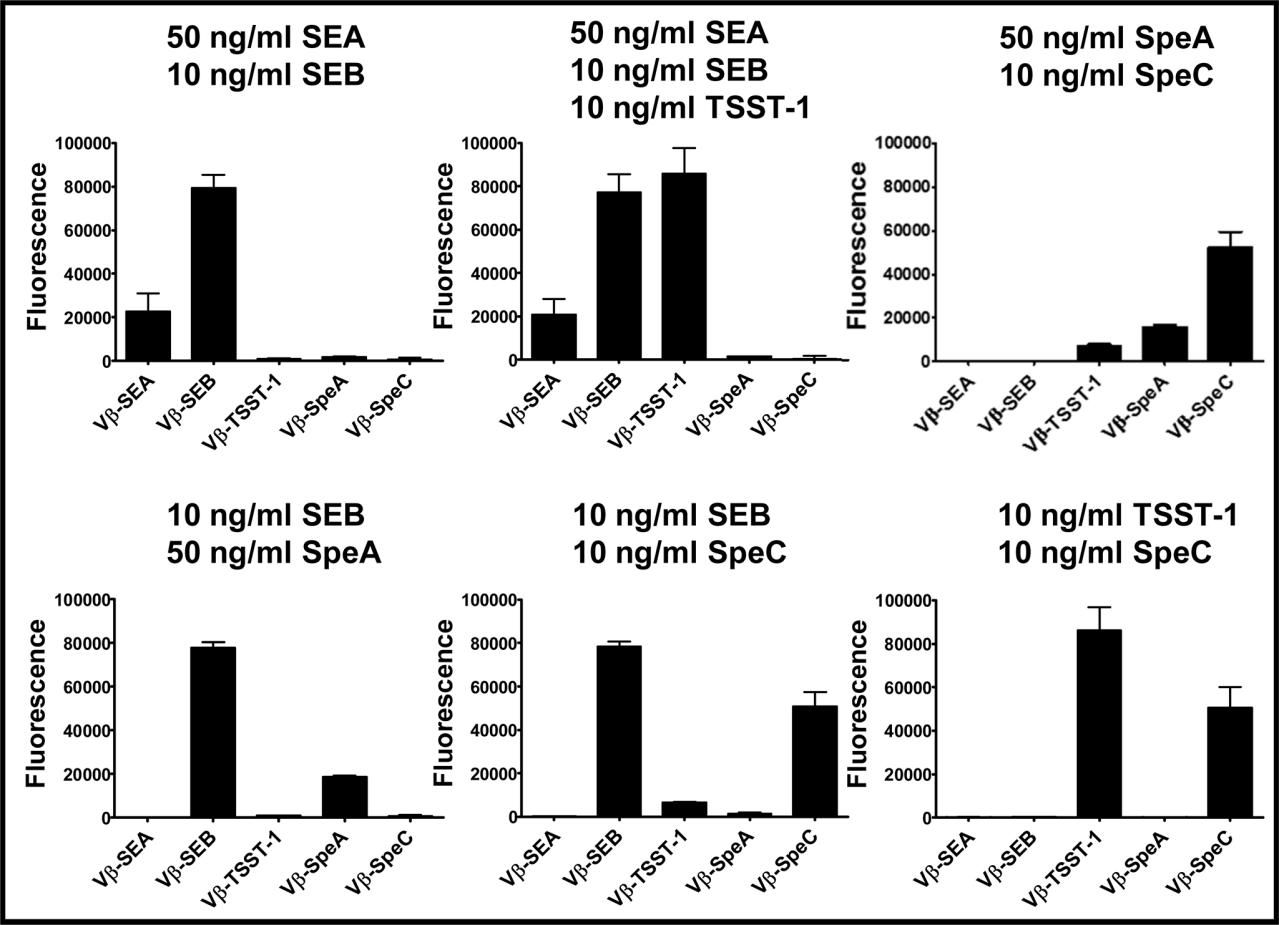
Multiplex assay in the presence of a mixture of toxins. Solutions containing a mixture of two or more toxins were tested in multiplex assays, at the indicated concentrations. Fluorescence emitted by each Vβ-immobilized bead due to toxin binding, was plotted on bar graphs shown. The error-bars represent standard deviations from two independent experiments. Similar data (but with higher background) were obtained in other experiments where unoccupied sites on biotin-Vβ immobilized-beads were not blocked with excess biotin (S5 Fig).

Various combinations of two or more toxins were also assayed in the multiplex format (Fig 5). Although the maximum fluorescence intensity observed with individual detection system varied, the assay showed that in all cases the expected toxin was detected in the appropriate channel for that Vβ-immobilized bead. We noted that the sensitivity of toxin detection in multiplex assay reduced by 1 to 2 orders of magnitude compared to the singleplex format, due to the higher background “noise” apparently associated with non-specific binding of Vβ-immobilized beads by the mixture of polyclonal antibodies.

### Multiplex assay for toxin detection in *Staphylococcus aureus* culture supernatants

**Fig 6:**
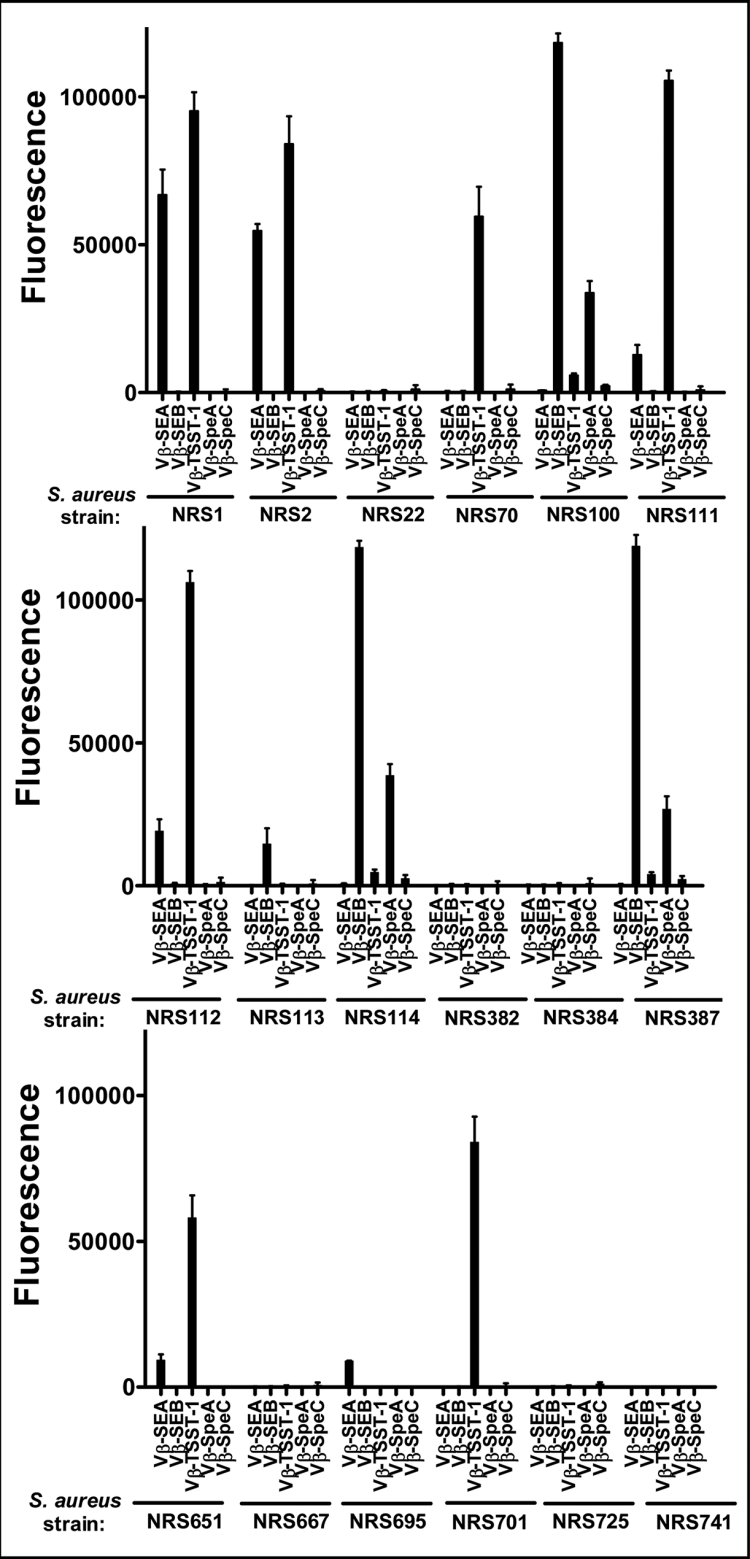
Multiplex assay with supernatants from cultures of various strains of *Staphylococcus aureus*. Supernatants (diluted 1:4) from cultures of 18 strains of *Staphylococcus aureus* obtained from the NARSA repository, were tested in a multiplex assay to determine their toxin expression profile. Fluorescence emitted by each Vβ-immobilized bead due to toxin binding, was plotted on bar graphs shown. Toxin(s) secreted by each strain was determined as an increase in fluorescence signal, compared to the background. The error-bars represent standard deviations from two independent experiments. Similar data (but with higher background) were obtained in other experiments where unoccupied sites on biotin-Vβ immobilized-beads were not blocked with excess biotin (S6 Fig).

To validate the assay for detection of toxins in potential clinical isolates or other unknown sources, we cultured 18 strains of *Staphylococcus aureus* from the NARSA repository. The strains were chosen based on the known SAg gene expression profiles of at least some of them. The multiplex assay was performed with diluted culture supernatants (diluted 1:4) from the 18 strains and the fluorescence intensity for each of the five Vβ systems was determined (Fig 6 and Table 1).

The results showed that 12 of the 18 strains expressed SEA, SEB and/or TSST-1 (note that some signal from Vβ-SpeA was present when SEB was detected due to its cross-reactivity with SEB). SEB was expressed in four of the *S*. *aureus* strains (NRS100, NRS113, NRS114 and NRS387). TSST-1 was expressed in seven strains (NRS1, NRS2, NRS70, NRS111, NRS112, NRS651, and NRS701). Interestingly, five of the seven TSST-1 positive strains (NRS1, NRS2, NRS111, NRS112 and NRS651) also expressed significant levels of SEA.

## Discussion

Serious clinical effects of the superantigens produced by *Staphylococcus aureus* and *Streptococcus pyogenes* have prompted considerable effort in developing assays for their detection in food sources or in clinical samples. The approaches have included DNA-based systems such as PCR for gene detection and use of antibodies as probes for detection of the toxins themselves. While PCR can provide a rapid and sensitive method for confirming the presence of genes encoding the toxin, this approach requires an adequate number of bacterial cells and it does not provide information about the level of the toxins that are present in a sample. For example, tissue-localized organisms could serve as a reservoir for the toxins that are released into the blood stream. Alternatively, cultures derived from a sterilized food source may be negative but the sample could contain preformed, heat stable enterotoxins. Hence, we propose that methods detecting the clinically relevant agent, the toxins, are a preferred approach.

In this study, highly sensitive, singleplex assays were developed for detection of five staphylococcal and streptococcal toxins that have been associated with human disease. The assays made use of high-affinity, *in vitro* engineered probes (a variable domain of the TCR β chain) for each of the toxins. The singleplex assays were combined to yield a multiplex assay that allowed reliable detection of the toxins in a single sample, including from culture supernatants of various *S*. *aureus* strains.

The design of the singleplex assays involved the arraying of high-affinity Vβ proteins, using site-directed biotinylation. The streptavidin-bound Vβ domains served as a capturing agent for the toxin against which they were engineered [32–34, 37]. Commercially available, polyclonal antibodies against the toxins were used as detecting agents.

By way of comparison, the assay used in the present study with biotinylated Vβ-SEB coated streptavidin beads enabled detection of SEB at concentrations as low as 3 pg/ml in singleplex assay. This was 40 fold better than our previous effort of detecting SEB where Vβ-SEB was coated on paramagnetic beads by means of amine coupling [31]. We believe that the site-specific biotinylation of Vβ protein allowed its immobilization on streptavidin coated in an optimal orientation hence leading to improved sensitivity of toxin detection, than immobilizing Vβ via amine coupling methods which may have yielded heterogeneous orientations.

Another factor that we believe contributed to the high sensitivity of SEB detection in singleplex assay was the remarkably high affinity of the engineered Vβ-SEB protein for SEB (K_D_ = 50 pM) [33]. In fact, the detection sensitivities for the other toxins in the singleplex assays, i.e. TSST-1 (25 pg/ml), SpeC (100 pg/ml), SpeA (6300 pg/ml) and SEA (400 pg/ml) also appeared to correlate to some extent with the affinities of the Vβ domains for their respective toxins: Vβ-TSST-1 (K_D_ = 50 pM), Vβ-SpeC (K_D_ = 500 pM), Vβ-SpeA (K_D_ = 270 pM), and Vβ-SEA (K_D_ = 4 nM) [32, 34, 35]. We suggest that the exception, lower sensitivity of SpeA detection despite the relatively high affinity of the Vβ-SpeA, was due to lack of a good, anti-SpeA polyclonal antibody, requiring us to use a cross-reacting anti-SEB polyclonal antibody for SpeA detection.

In addition to the contribution to the sensitivity of the singleplex assays, the use of two distinct toxin binding proteins (i.e., high-affinity Vβ proteins to capture the toxin and anti-toxin antibody to detect the “captured” toxin) provided additional specificity to the toxin detection. In multiplex assay, some expected cross-reactivities identified in the singleplex format, were noted for high-affinity Vβ proteins which were engineered from same template Vβ (Vβ-SpeA and Vβ-SEB engineered from mouse Vβ8 cross-reacted with SEB and SpeA respectively; Vβ-SpeC and Vβ-TSST-1 engineered from human Vβ2.1, cross-reacted with TSST-1 and SpeC respectively) (S3 Fig) [32, 34].

The flow cytometry-based assays described here can be used to detect toxins in food sources or in culture supernatants of *Staphylococcus aureus* and/or *Streptococcus pyogenes*, or in clinical samples. Enterotoxins like SEA in contaminated food sources have been shown to be present in the range from 0.05 to 20 ng/g of food [29, 42]. In food samples from food poisoning outbreaks, staphylococcal enterotoxins (particularly, SEA, SEE and SEH) were reported in the range of 0.08 to 0.5 ng/ml [4, 5] and 0.36–20 ng/g [4, 7, 8, 12]. Importantly, these studies required concentration steps of the food sample in order to detect and quantitate the levels of the toxins that were recovered. These assays reported assay sensitivities from ~ 0.1–0.5 ng/ml, similar to our singleplex assay for SEA (0.4 ng/ml SEA). However, our SEB and TSST-1 assays had sensitivities that were one to two orders of magnitude higher than these and other commercial assays [11, 25, 43, 44]. The possible linkage between SEA and TSST-1 expression among strains suggests that TSST-1 detection could be a biomarker for SEA, if the latter falls below the levels of detection. As the TSST-1 singleplex assay developed here could detect 25 pg/ml, we suggest that it could be used directly for sampling food sources without the need for a concentration step, but the biotin-Vβ-based assays will now need to be tested using various food matrices.

More recently, highly automated, DNA based tests have been developed for rapid identification of microorganisms from blood cultures of patients [45, 46]. Since these tests also provide information about antibiotic resistance of the microorganism present, they could guide appropriate antibiotic treatment for patients. However in situations where a patient’s blood culture is negative for microorganisms, for example, in a case of deep tissue infection by *Staphylococcus aureus* or *Streptococcus pyogenes*, the patient could still develop shock-like or inflammatory symptoms that might arise due to the secretion of superantigenic toxins by the bacteria [47–49]. In such situations, or in cases when bacterial burden in blood is minimal, methods that detect the toxin directly could be useful.

In this study, 18 strains of *Staphylococcus aureus* obtained from the NARSA repository were also examined for toxin secretion. The collection included several strains that were isolated from clinical samples. The supernatants from these cultures were analyzed by multiplex assay for simultaneous detection of SEA, SEB and TSST-1. The expression of the toxins was consistent with the presence of genes in strains for which genomic data was available, as in the case of NRS1 (*entA* (SEA) and *tst* (TSST-1)), NRS70 (*tst* (TSST-1)) and NRS100 (*entB* (SEB)). NRS22 served as a negative control since it has been reported to lack the genes that encode SEA, SEB, and TSST-1. Complete absence of signal in five other strains (NRS382, NRS384, NRS667, NRS725, NRS741) supported the view that the assay was specific for the target toxins and did not yield false positives for unrelated proteins, such as Protein A expressed by *S*. *aureus* [25, 28].

We also noted that five of the seven strains that expressed TSST-1 also expressed SEA. This toxin expression profile was particularly interesting because previous reports have linked SEA co-expression with TSST-1 in menstrual TSS, more often than in non-menstrual TSS [50, 51]. In our collection of strains, NRS112 (MN8) was isolated from a menstrual TSS patient and we detected both SEA and TSST-1 in the culture supernatant of this strain. Interestingly, other investigators detected the gene corresponding to SEA in this strain by PCR, but they could not detect the protein probably because the concentration of SEA secreted was very low [38]. The *S*. *aureus* strain NRS111 (FRI913) was obtained from a food poisoning outbreak, and it was thus not surprising that SEA was detected in the culture supernatant of this strain, since SEA has been incriminated in most *S*. *aureus* food poisoning outbreaks around the world [4, 5, 7–9]. We also detected TSST-1 which, although not enterotoxic, could serve as a biomarker for the presence of *S*. *aureus* or possibly even SEA [52]. Investigators have confirmed the presence of *sea* and *tst* genes in this strain by PCR [39]. Our detection of SEB in NRS114 was also consistent with the presence of the *seb* gene by PCR [39].

In summary, we present here a sensitive flow cytometry-based assay for the detection of three staphylococcal and two streptococcal toxins using ultra-high-affinity Vβ proteins and polyclonal antibody reagents. Similar approaches can be used for the detection of other SAgs. Multiplex formatting of the assays, using beads with a range of fluorescent dyes, enabled simultaneous detection of SEA, SEB and TSST-1, in culture supernatants of several strains of *S*. *aureus*. We also reported the multiplex assay could be used for detection of SpeA and SpeC toxins, for which no commercial assays are available yet.

## Supporting Information

S1 Fig Pilot, singleplex assays for assessing binding of high-affinity Vβ proteins to the toxins against which they were engineered. Biotinylated, high-affinity Vβ proteins (Vβ-SEA, Vβ-SEB, Vβ-TSST-1, Vβ-SpeA and Vβ-SpeC) immobilized on individual streptavidin-coated fluorescent beads (P12, P10, P6, P2 and P8 respectively), were incubated with 50 nM toxin against which they were engineered (SEA, SEB, TSST-1, SpeA and SpeC respectively). Toxins bound by the Vβ-immobilized beads were detected by rabbit polyclonal anti-toxin antibodies (anti-SEA, anti-SEB, anti-TSST-1, anti-SpeA and anti-SpeC respectively) followed by goat-anti rabbit IgG labeled with Alexa fluor 647. Flow cytometry histograms indicating fluorescence emitted by the beads themselves (FL-1 channel, left panel), and due to bound toxin (FL-4 channel, right panel) are shown. Fluorescence in the absence of toxin, is represented by gray (filled) trace on each histogram.
(TIFF)

S2 Fig Capture ELISA for toxin detection. High-affinity Vβ proteins were immobilized on wells of an ELISA plate to capture various concentrations of cognate, recombinant toxins. Bound toxins were detected by polyclonal, anti-toxin antibodies from rabbit, followed by goat-anti rabbit IgG-HRP. TMB substrate was added and the reaction was stopped with 1N H_2_SO_4_ to yield a yellow colored product. Absorbance at 450 nm was recorded and used to generate binding curves. Red, dashed line indicates absorbance in the absence of toxin. The error-bars represent standard deviations from two independent experiments.
(TIF)

S3 Fig Cross-reactivity of related Vβ with non-cognate toxins in multiplex assay. Solutions containing different concentrations of various toxins were tested in multiplex assays. Fluorescence emitted by each Vβ-immobilized bead due to toxin binding, was plotted on bar graphs: Vβ-SEA (purple), Vβ-SEB (orange), Vβ-TSST-1 (blue), Vβ-SpeA (red) and Vβ-SpeC (green). Vβ-SpeA (red) cross-reacted with SEB, Vβ-SpeC (green) cross-reacted with TSST-1 and Vβ-TSST-1(blue) cross-reacted with SpeC. The error-bars represent standard deviations from two independent experiments.
(TIFF)

S4 Fig Effect of blocking Vβ-immobilized beads with biotin on multiplex assay in the presence of one toxin. Solutions containing different concentrations of various toxins were tested in multiplex assays, in the presence of Vβ-immobilized beads that were incubated in the absence or presence of biotin (~two-fold or thousand-fold excess biotin was added, compared to the biotin-binding sites available on the beads). Fluorescence emitted by each Vβ-immobilized bead due to toxin binding, was plotted on bar graphs: Vβ-SEA (purple), Vβ-SEB (orange), Vβ-TSST-1 (blue), Vβ-SpeA (red) and Vβ-SpeC (green).
(TIFF)

S5 Fig Effect of blocking Vβ-immobilized beads with biotin on multiplex assay in the presence of a mixture of toxins. Solutions containing a mixture of two or more toxins were tested in multiplex assays, in the presence of Vβ-immobilized beads that were incubated in the absence or presence of biotin (~1000-fold excess biotin was added, compared to the biotin-binding sites available on the beads). Fluorescence emitted by each Vβ-immobilized bead due to toxin binding, was plotted on bar graphs: Vβ-SEA (purple), Vβ-SEB (orange), Vβ-TSST-1 (blue), Vβ-SpeA (red) and Vβ-SpeC (green).
(TIFF)

S6 Fig Effect of blocking Vβ-immobilized beads with biotin on multiplex assay in the presence of supernatants from cultures of various strains of *Staphylococcus aureus*. Supernatants (diluted 1:4) from cultures of 18 strains of *Staphylococcus aureus* obtained from the NARSA repository, were tested in multiplex assays to determine their toxin expression profile. The assays were performed with Vβ-immobilized beads that were incubated in the absence or presence of biotin (~1000-fold excess biotin was added, compared to the biotin-binding sites available on the beads). Fluorescence emitted by each Vβ-immobilized bead due to toxin binding, was plotted on bar graphs: Vβ-SEA (purple), Vβ-SEB (orange), Vβ-TSST-1 (blue), Vβ-SpeA (red) and Vβ-SpeC (green).
(TIFF)
