# Supplementary figures and images for: A Multiplex Assay for Detection of Staphylococcal and Streptococcal Exotoxins
Source: PLoS One. 2015 Aug 25;10(8):e0135986. doi: 10.1371/journal.pone.0135986 (PMC4549143; doi:10.1371/journal.pone.0135986)

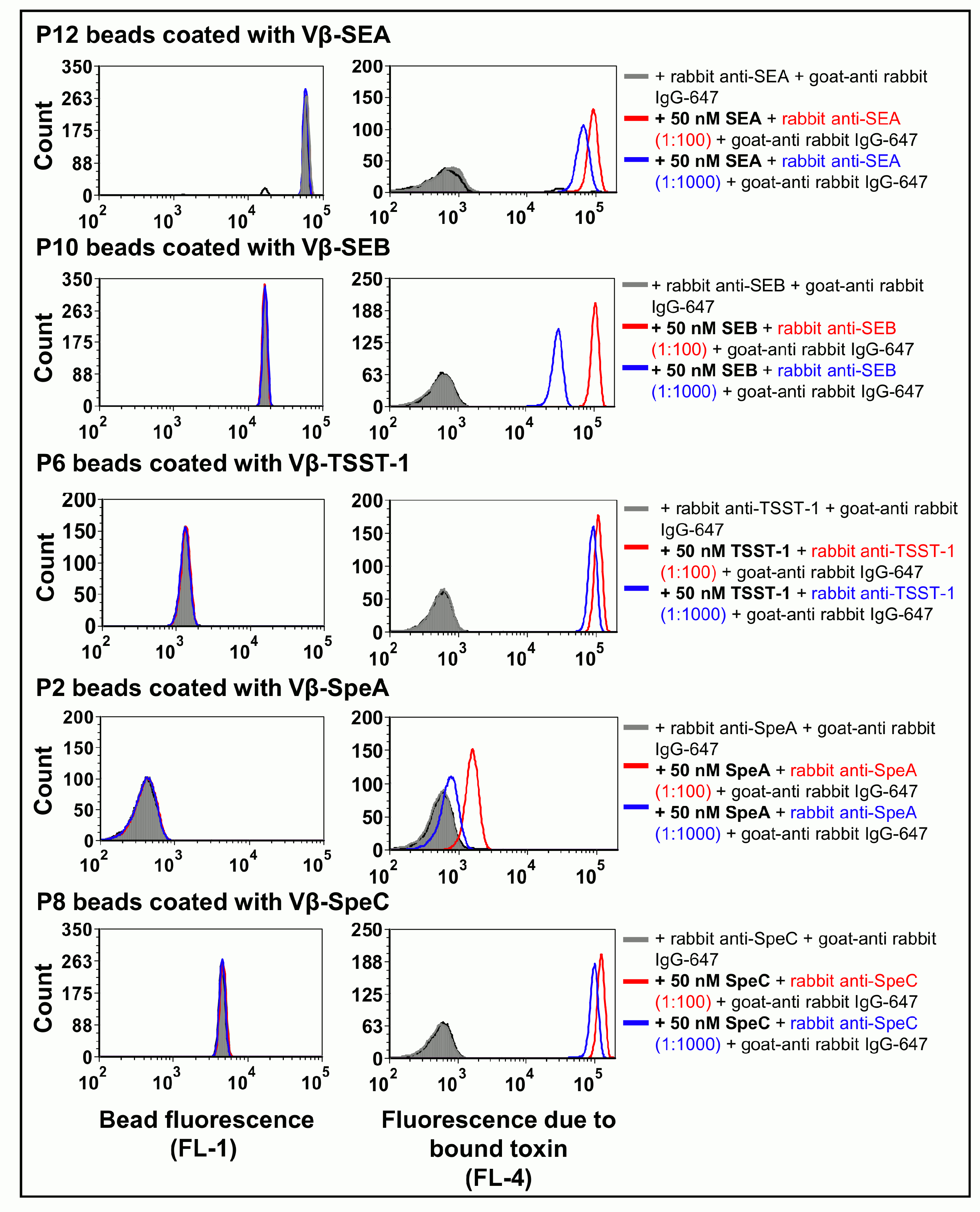

Supplement: S1 Fig — Biotinylated, high-affinity Vβ proteins (Vβ-SEA, Vβ-SEB, Vβ-TSST-1, Vβ-SpeA and Vβ-SpeC) immobilized on individual streptavidin-coated fluorescent beads (P12, P10, P6, P2 and P8 respectively), were incubated with 50 nM toxin against which they were engineered (SEA, SEB, TSST-1, SpeA and SpeC respectively). Toxins bound by the Vβ-immobilized beads were detected by rabbit polyclonal anti-toxin antibodies (anti-SEA, anti-SEB, anti-TSST-1, anti-SpeA and anti-SpeC respectively) followed by goat-anti rabbit IgG labeled with Alexa fluor 647. Flow cytometry histograms indicating fluorescence emitted by the beads themselves (FL-1 channel, left panel), and due to bound toxin (FL-4 channel, right panel) are shown. Fluorescence in the absence of toxin, is represented by gray (filled) trace on each histogram. (TIFF) [file pone.0135986.s001.tiff]

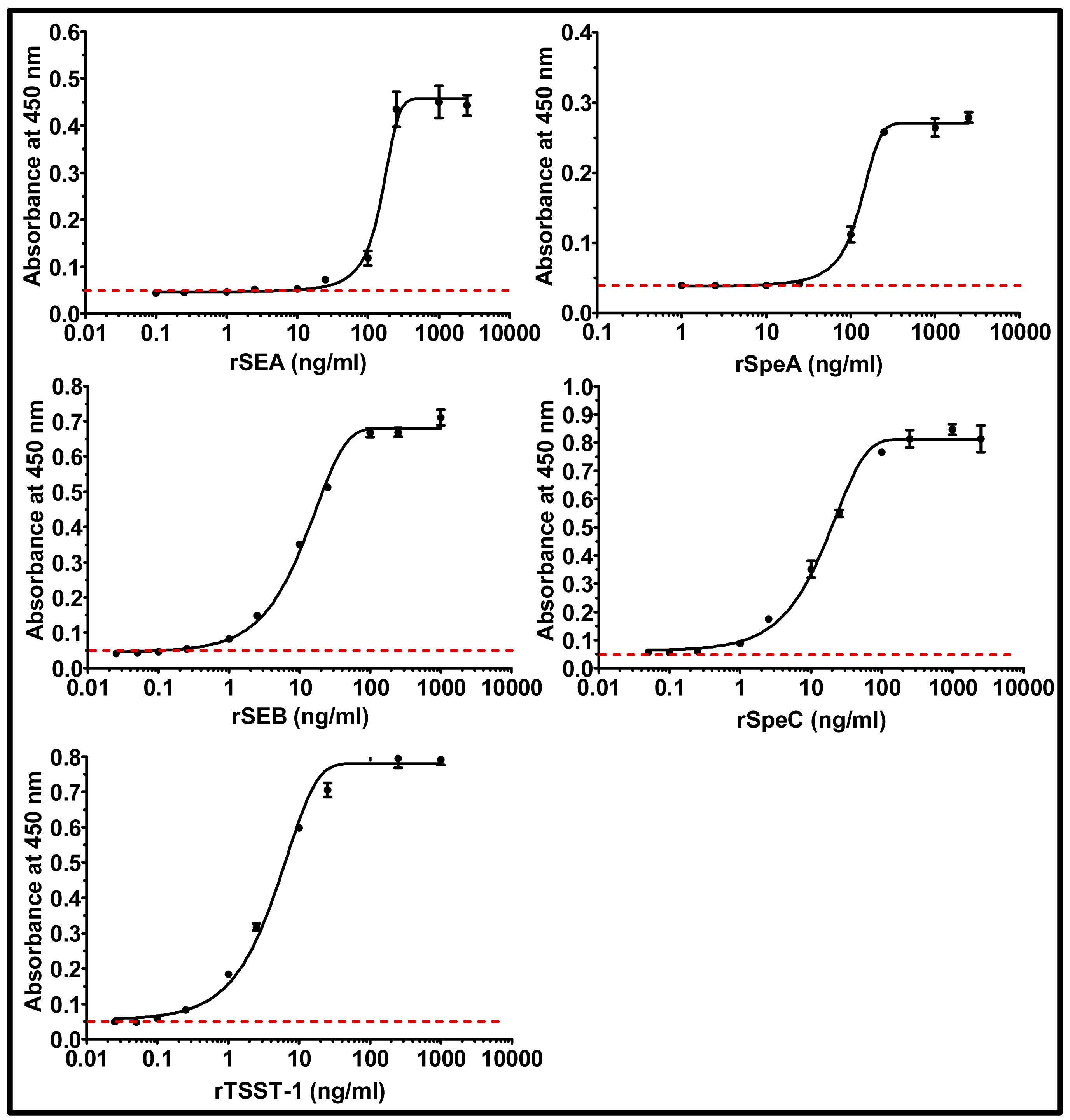

Supplement: S2 Fig — High-affinity Vβ proteins were immobilized on wells of an ELISA plate to capture various concentrations of cognate, recombinant toxins. Bound toxins were detected by polyclonal, anti-toxin antibodies from rabbit, followed by goat-anti rabbit IgG-HRP. TMB substrate was added and the reaction was stopped with 1N H2SO4 to yield a yellow colored product. Absorbance at 450 nm was recorded and used to generate binding curves. Red, dashed line indicates absorbance in the absence of toxin. The error-bars represent standard deviations from two independent experiments. (TIF) [file pone.0135986.s002.tif]

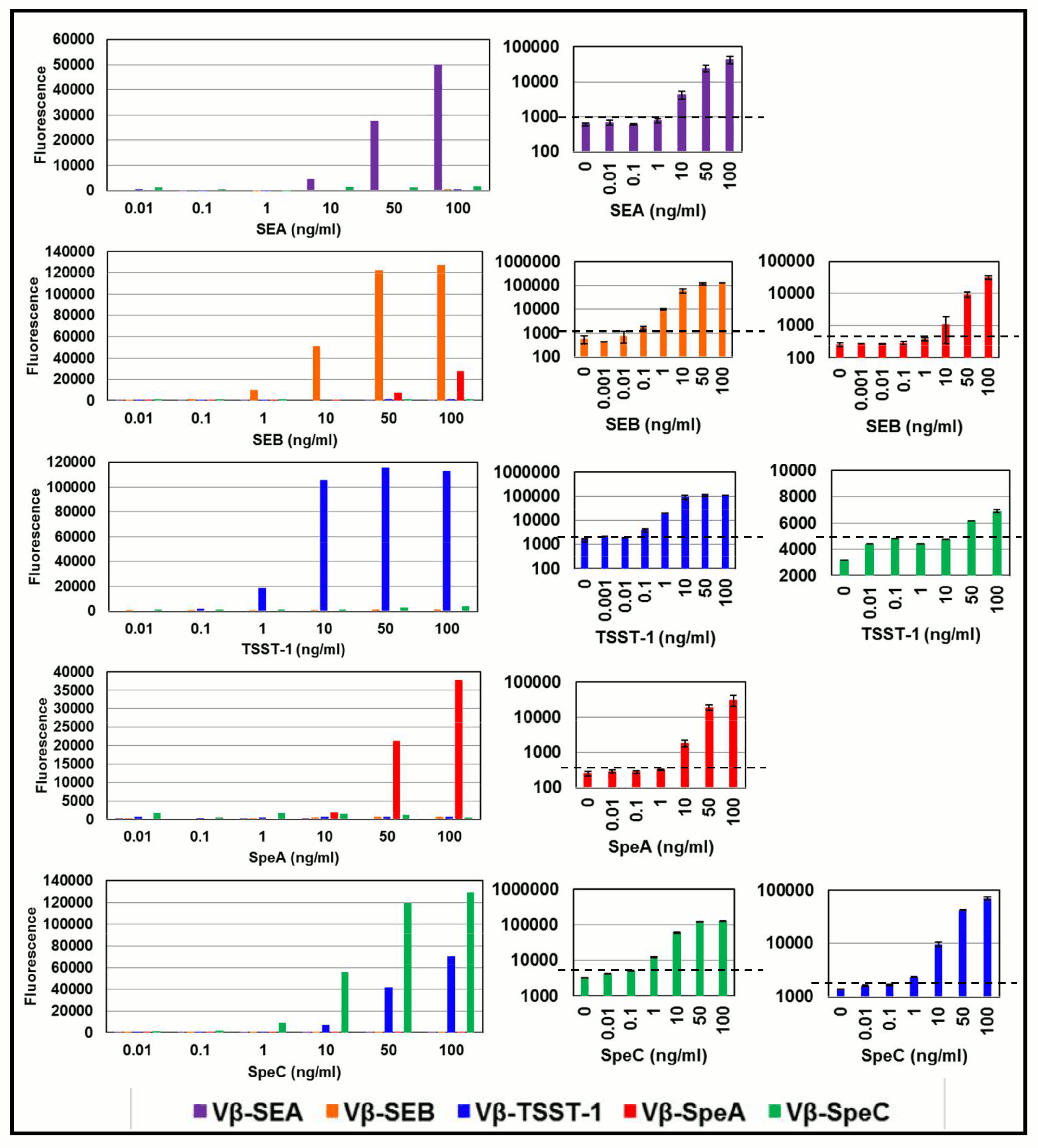

Supplement: S3 Fig — Solutions containing different concentrations of various toxins were tested in multiplex assays. Fluorescence emitted by each Vβ-immobilized bead due to toxin binding, was plotted on bar graphs: Vβ-SEA (purple), Vβ-SEB (orange), Vβ-TSST-1 (blue), Vβ-SpeA (red) and Vβ-SpeC (green). Vβ-SpeA (red) cross-reacted with SEB, Vβ-SpeC (green) cross-reacted with TSST-1 and Vβ-TSST-1(blue) cross-reacted with SpeC. The error-bars represent standard deviations from two independent experiments. (TIFF) [file pone.0135986.s003.tiff]

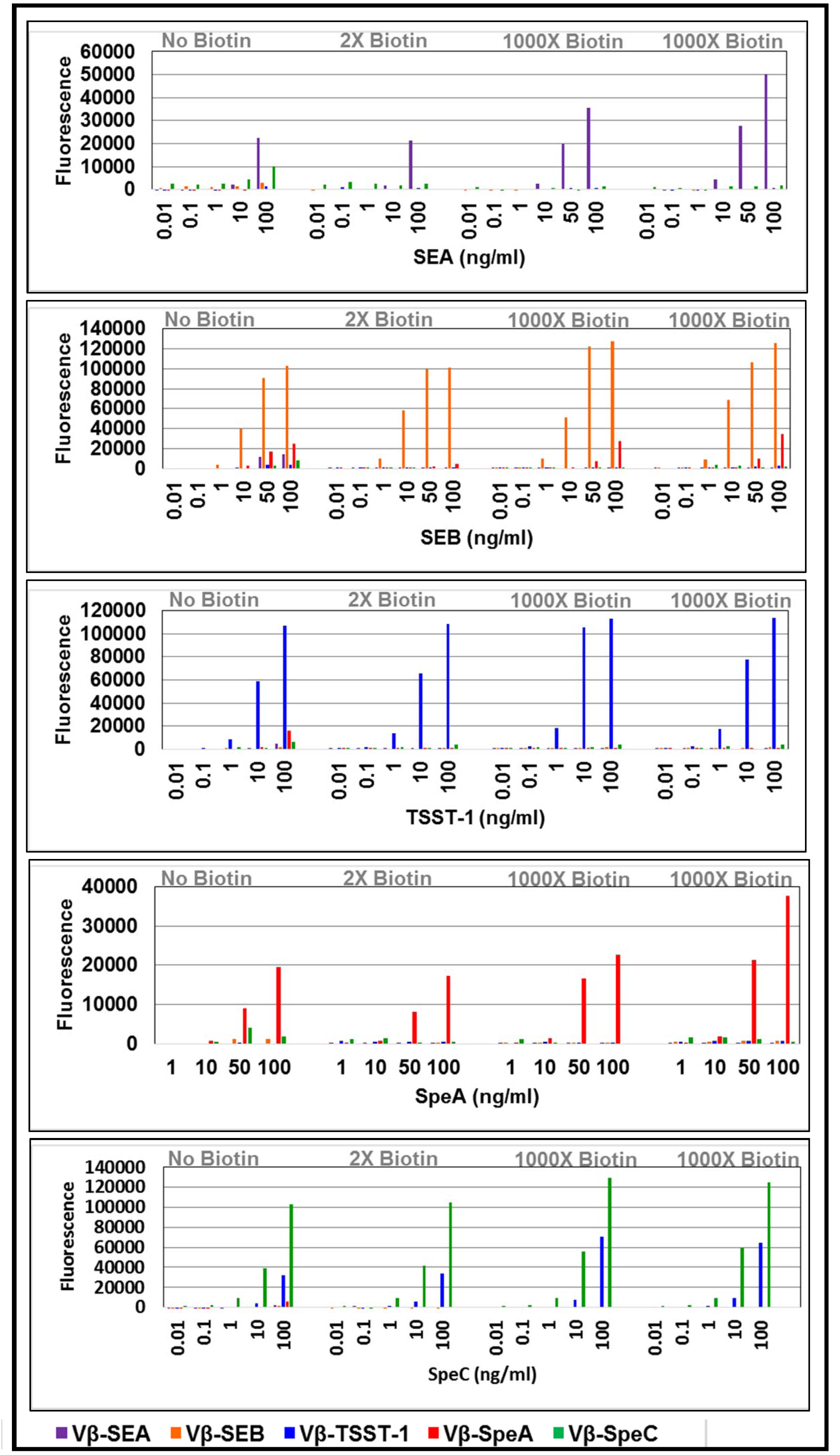

Supplement: S4 Fig — Solutions containing different concentrations of various toxins were tested in multiplex assays, in the presence of Vβ-immobilized beads that were incubated in the absence or presence of biotin (~two-fold or thousand-fold excess biotin was added, compared to the biotin-binding sites available on the beads). Fluorescence emitted by each Vβ-immobilized bead due to toxin binding, was plotted on bar graphs: Vβ-SEA (purple), Vβ-SEB (orange), Vβ-TSST-1 (blue), Vβ-SpeA (red) and Vβ-SpeC (green). (TIFF) [file pone.0135986.s004.tiff]

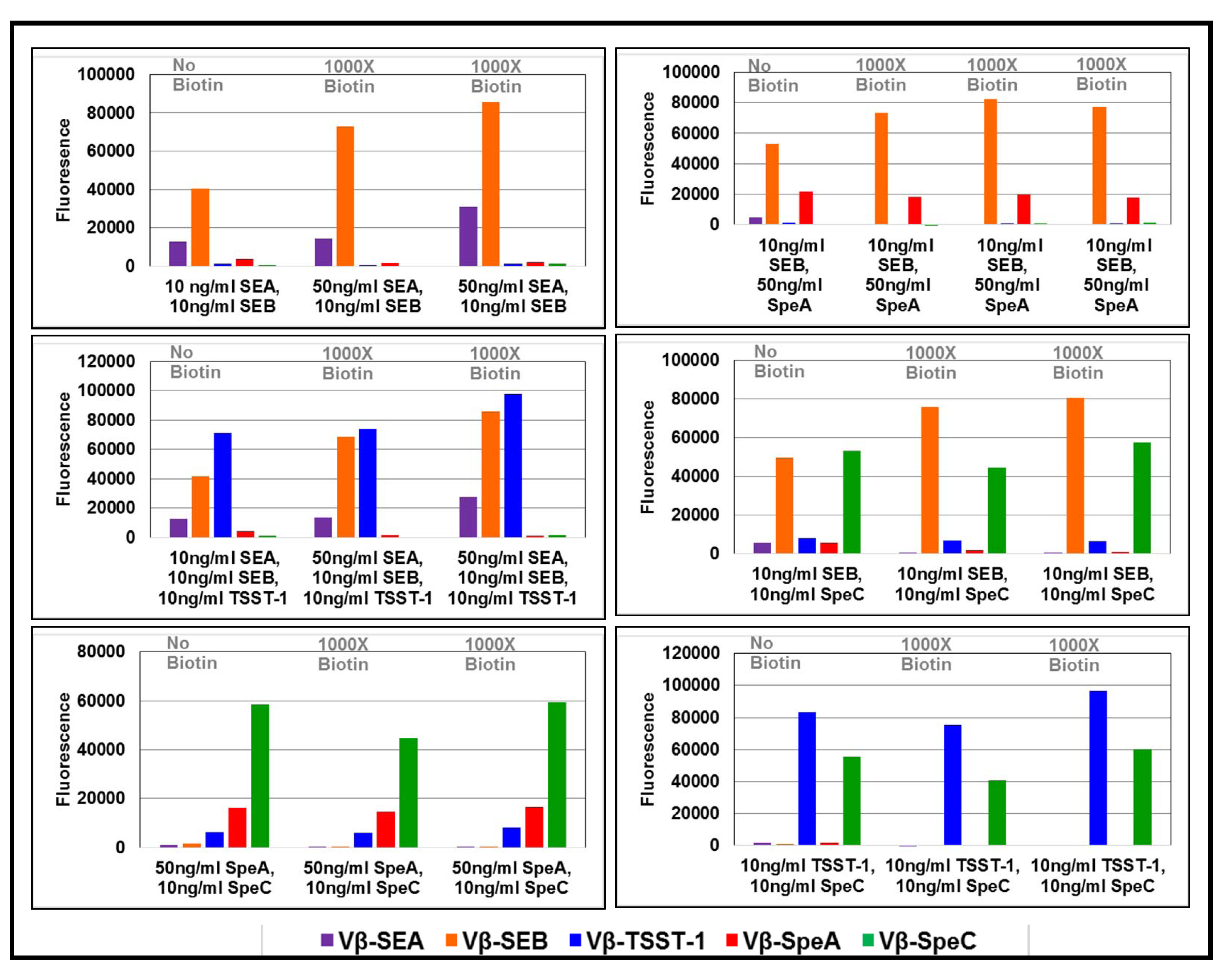

Supplement: S5 Fig — Solutions containing a mixture of two or more toxins were tested in multiplex assays, in the presence of Vβ-immobilized beads that were incubated in the absence or presence of biotin (~1000-fold excess biotin was added, compared to the biotin-binding sites available on the beads). Fluorescence emitted by each Vβ-immobilized bead due to toxin binding, was plotted on bar graphs: Vβ-SEA (purple), Vβ-SEB (orange), Vβ-TSST-1 (blue), Vβ-SpeA (red) and Vβ-SpeC (green). (TIFF) [file pone.0135986.s005.tiff]

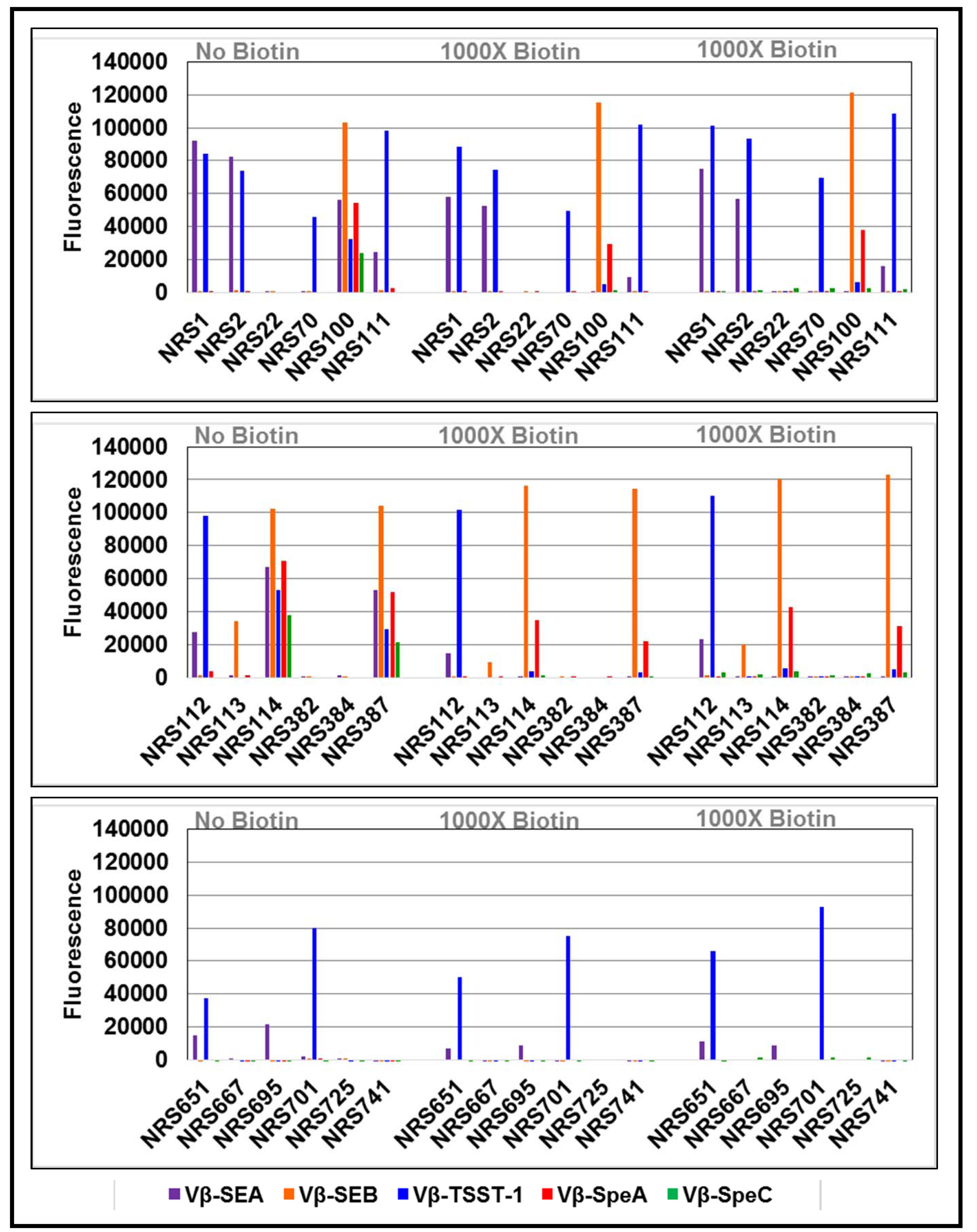

Supplement: S6 Fig — Supernatants (diluted 1:4) from cultures of 18 strains of Staphylococcus aureus obtained from the NARSA repository, were tested in multiplex assays to determine their toxin expression profile. The assays were performed with Vβ-immobilized beads that were incubated in the absence or presence of biotin (~1000-fold excess biotin was added, compared to the biotin-binding sites available on the beads). Fluorescence emitted by each Vβ-immobilized bead due to toxin binding, was plotted on bar graphs: Vβ-SEA (purple), Vβ-SEB (orange), Vβ-TSST-1 (blue), Vβ-SpeA (red) and Vβ-SpeC (green). (TIFF) [file pone.0135986.s006.tiff]
